# Supplementary figures and images for: Development and utilization of a surrogate SARS-CoV-2 viral neutralization assay to assess mRNA vaccine responses
Source: PLoS One. 2022 Jan 18;17(1):e0262657. doi: 10.1371/journal.pone.0262657 (PMC8765639; doi:10.1371/journal.pone.0262657)

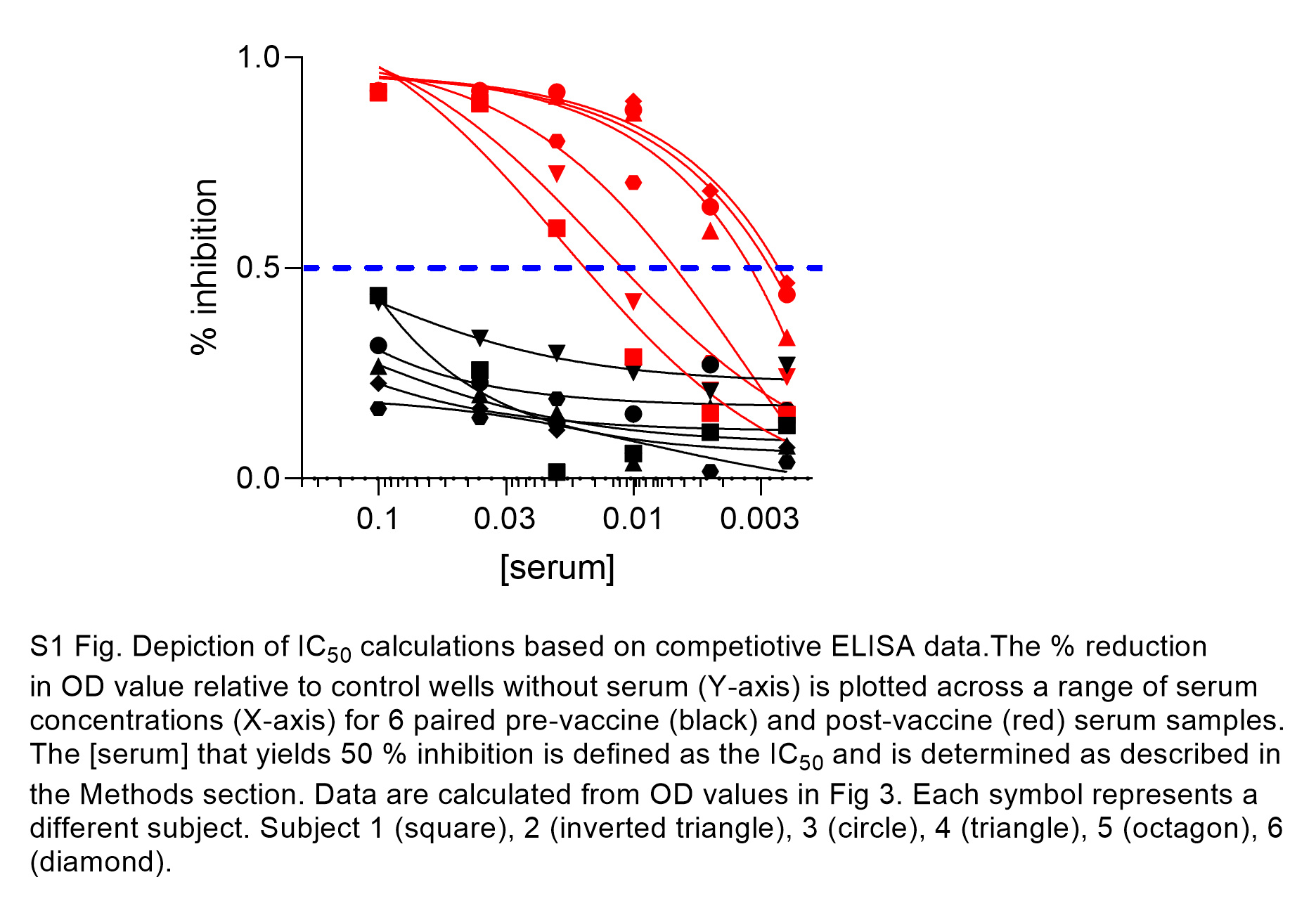

Supplement: S1 Fig — (TIF) [file pone.0262657.s001.tif]

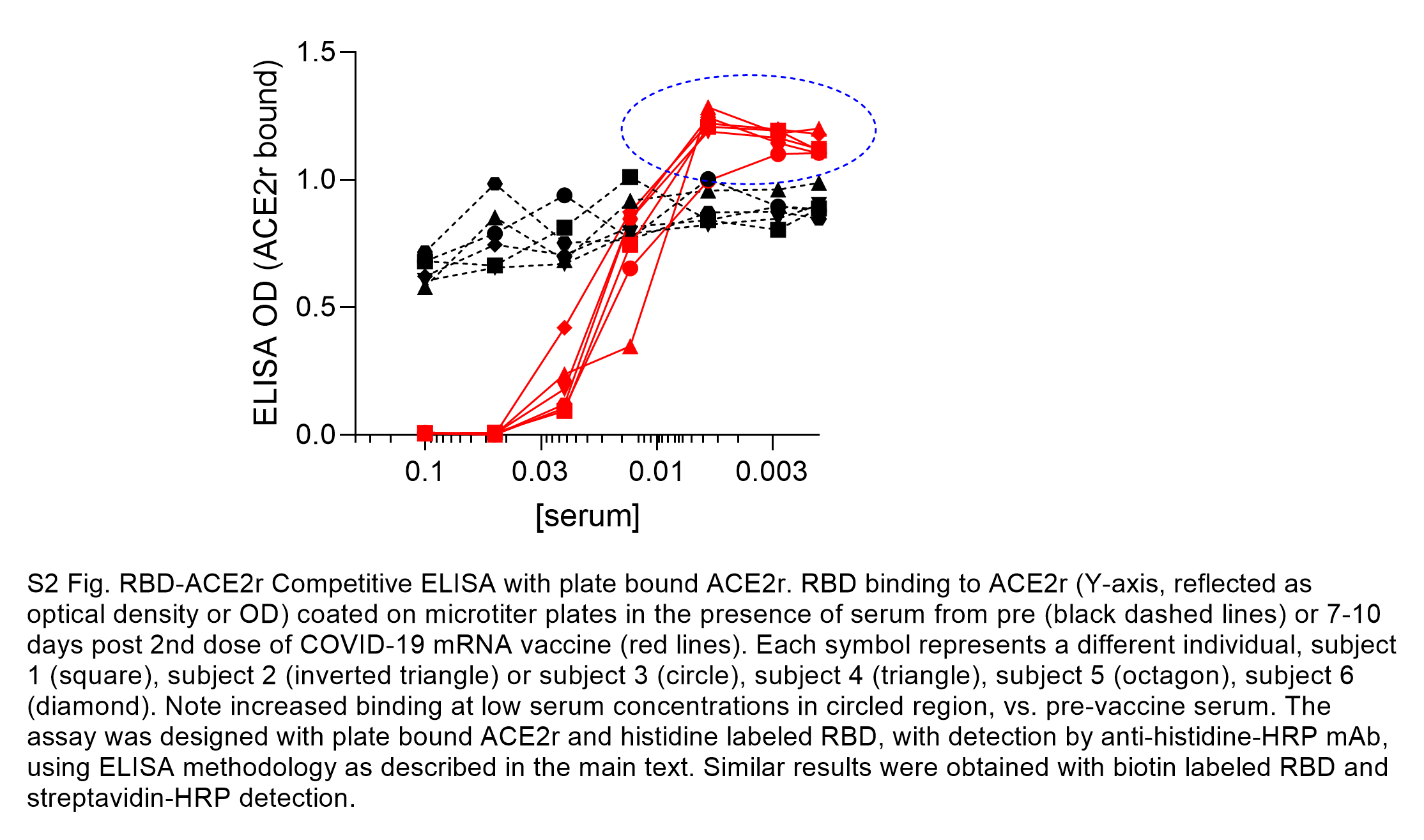

Supplement: S2 Fig — (TIF) [file pone.0262657.s002.tif]
