# Supplementary material for: Development and utilization of a surrogate SARS-CoV-2 viral neutralization assay to assess mRNA vaccine responses
Source: PLoS One. 2022 Jan 18;17(1):e0262657. doi: 10.1371/journal.pone.0262657 (PMC8765639; doi:10.1371/journal.pone.0262657)
Supplement: S3 Fig — (PDF) [file pone.0262657.s003.pdf]

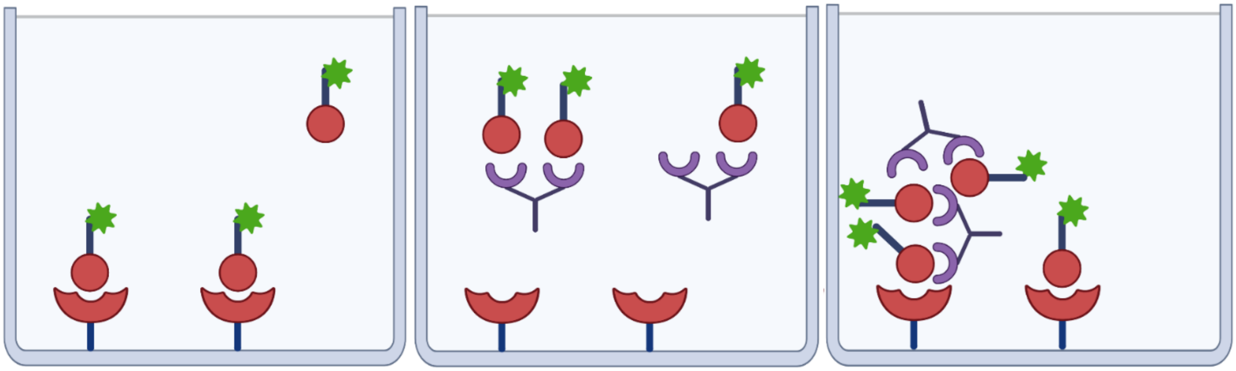

*S3 Fig. Schematic depiction of potential RBD cross-linkage in competitive ELSA. Labeled RBD is depicted as *ball and stick and ACE2r is depicted bound to plate. Middle and right show antibody blocking binding or cross-linking RBD to ACE2r respectively.*
