# Supplementary material for: Development and utilization of a surrogate SARS-CoV-2 viral neutralization assay to assess mRNA vaccine responses
Source: PLoS One. 2022 Jan 18;17(1):e0262657. doi: 10.1371/journal.pone.0262657 (PMC8765639; doi:10.1371/journal.pone.0262657)
Supplement: S4 Fig — (PDF) [file pone.0262657.s004.pdf]

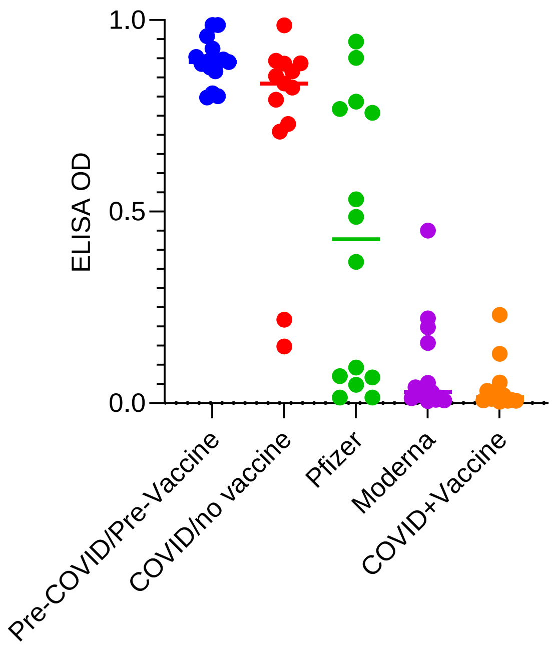

S4 Fig. Inhibition of ACE2r binding to RBD by serum (1:25 dilution) from different groups of individuals. The ability of different groups of individuals serum (described in Table 1 and Fig 5) to inhibit ACE2r binding to RBD was quantified. The ELISA OD (Y-axis), reflecting ACE2r binding was determined in the presence of a 1:25 dilution of serum. Each symbol represents a different subject.
